# In Situ Accumulation of CaOx Crystals in *C. quitensis* Leaves and Its Relationship with Anatomy and Gas Exchange

**DOI:** 10.3390/plants13060769

**Published:** 2024-03-08

**Authors:** Olman Gómez-Espinoza, Francisca I. Fuentes, Constanza F. Ramírez, León A. Bravo, Patricia L. Sáez

**Affiliations:** 1 Laboratorio de Fisiología y Biología Molecular Vegetal, Departamento de Ciencias Agronómicas y Recursos Naturales, Facultad de Ciencias Agropecuarias y Medioambiente, Universidad de La Frontera, Temuco 4811230, Chile; olman.gomez@ufrontera.cl (O.G.-E.); francisfuentese@gmail.com (F.I.F.); leon.bravo@ufrontera.cl (L.A.B.); 2 Laboratorio Cultivo de Tejidos Vegetales, Centro de Biotecnología, Departamento de Silvicultura, Facultad de Ciencias Forestales, Universidad de Concepción, Casilla 160-C, Concepción 4030000, Chile; consramirez@udec.cl; 3 Instituto de Ecología y Biodiversidad—IEB, Las Palmeras 3425, Ñuñoa, Santiago 7800003, Chile

**Keywords:** alarm photosynthesis, CaOX crystals, *C. quitensis*, mesophyll conductance, photosynthesis

## Abstract

The accumulation of crystal calcium oxalate (CaOx) in plants is linked to a type of stress-induced photosynthesis termed ‘alarm photosynthesis’, serving as a carbon reservoir when carbon dioxide (CO_2_) exchange is constrained. *Colobanthus quitensis* is an extremophyte found from southern Mexico to Antarctica, which thrives in high-altitude Andean regions. Growing under common garden conditions, *C. quitensis* from different latitudinal provenances display significant variations in CaOx crystal accumulation. This raises the following questions: are these differences maintained under natural conditions? And is the CaOx accumulation related to mesophyll conductance (g_m_) and net photosynthesis (A_N_) performed in situ? It is hypothesized that in provenances with lower g_m_, *C. quitensis* will exhibit an increase in the use of CaOx crystals, resulting in reduced crystal leaf abundance. Plants from Central Chile (33°), Patagonia (51°), and Antarctica (62°) were measured in situ and sampled to determine gas exchange and CaOx crystal accumulation, respectively. Both A_N_ and g_m_ decrease towards higher latitudes, correlating with increases in leaf mass area and leaf density. The crystal accumulation decreases at higher latitudes, correlating positively with A_N_ and g_m_. Thus, in provenances where environmental conditions induce more xeric traits, the CO_2_ availability for photosynthesis decreases, making the activation of alarm photosynthesis feasible as an internal source of CO_2_.

## 1. Introduction

The sustained changes in environmental conditions at the global level have increased the need to understand how plant species respond and adjust to these changes [1]. One approach to studying these responses is analyzing the plant performance along latitudinal gradients, which offer a diverse range of environmental conditions, acting as natural laboratories [1]. This approach could be particularly interesting in ecosystems where climate change is already evident, such as high mountain environments and Antarctica [2]. *Colobanthus quitensis* Kunt Bartl (Cariophyllaceae) is an extremophile plant species naturally distributed in the Andean-Patagonian-Antarctic gradient [3], and is mainly associated with high mountain and harsh environmental conditions [4]. Given its wide geographical distribution and the isolation of their populations, Andean and Antarctic ecotypes for cold resistance have been defined in this species [4]. The *C. quitensis* ecotypes exhibit both morphological and genetic variability, likely attributed to continuous selection processes in response to the prevailing conditions at each habitat [5,6]. This variability could be linked to the plant capacity to adapt to harsh conditions, offering valuable insights into the underlying stress tolerance mechanisms. Overall, *C. quitensis* provenances constitute an excellent model for conducting latitudinal gradient studies by showing ecotypic differences and still having relatively high genetic similarity between populations [6,7].

Among the several mechanisms that *C. quitensis* deploys to overcome the harsh environmental conditions (see Ramirez et al. (2024) [8] and references therein), a trait that has recently received attention is the significant presence of calcium oxalate (CaOx) crystals in the leaves and the potential role of these structures in stress tolerance [9]. CaOx crystals are essential and multifunctional plant tools, their accumulation being exceptionally high in plants inhabiting harsh environments [10]. For instance, in rain forests, 15–53% of species show CaOx crystals in the leaves, and this percentage increases in montane forests (76–86%), being even higher in xerophytes species. Succulent and drought-resistant species in deserts have notable quantities of CaOx crystals, highlighting the strong association between CaOx crystals and stressful environments [10]. Under stressful conditions such as drought or carbon starvation, CaOx has been associated with the alarm photosynthesis process, where CaOx crystals act as a biochemical mechanism that stores carbon as oxalate, primarily during nighttime when the stomata are closed and photosynthesis is inactive. In daylight hours, the crystal decomposition contributes additional carbon for photosynthetic assimilation [11].

A previous study suggested that *C. quitensis* employs alarm photosynthesis as an inherent process when faced with CO_2_-limiting conditions. When *C. quitensis* was growing at low atmospheric CO_2_ concentration, the leaf CaOx crystal area decreased over time, which was accompanied by an increase in the oxalate oxidase activity and a slight decrease in the electron transport rate (ETR) [9]. Moreover, the observation of the leaf’s crystals for 24 h, under optimal growth conditions, showed that the crystal area remains stable during the initial hours of daylight but decreases significantly from 12:00 to 20:00 h. However, during the dark hours, the crystals fully recover. This suggests that crystals may be involved in supplementing the plant’s CO_2_ requirements, especially during daylight when photosynthesis is operating [9]. In addition, Antarctic *C. quitensis* plants deploy xerophytic traits such as high leaf density (LD), leaf mass area (LMA), and cell walls thickness (T_cw_), which reduce the CO_2_ availability through the mesophyll to the chloroplast. These traits serve as mechanisms to avoid water loss under the Antarctic field conditions which, consequently, results in a notably low mesophyll conductance (g*_m_*) for CO_2_ [12,13]. This further reinforces the possible role of alarm photosynthesis in *C. quitensis* since it has been predicted that the frequency and intensity of this process tend to be higher in xerophytes [11], and that plants with lower g*_m_* could use a significant proportion of CO_2_ from internal sources for photosynthesis, which may be partially provided by CaOx crystals [9]. This is also consistent with the paradox that even though *C. quitensis* from Antarctica manifest an extremely low g_m_, the net photosynthetic rates can reach relatively high values [12,13].

In addition, a prior study revealed that under common garden conditions, the *C. quitensis* provenances from the Antarctic and Central Chile (Andean) displayed noticeable morphological differences, including variations in habit size, leaf dimensions (length-width ratio), and significant differences in CaOx accumulation, despite being cultivated under identical conditions. While the differences found could be linked to processes of ecotypic differentiation and plant adaptation to harsh environments, this has not yet been proven [14]. Thus, although *C. quitensis* has shown evidence for the use of CaOx crystals as a carbon source, this evidence came only from studies under laboratory conditions, and the relationship with the photosynthetic performance at their natural habitat has yet to be studied. This raises the question if the differences in CaOx accumulation observed under common garden conditions are maintained under natural conditions in the latitudinal gradient, and if the CaOx crystal accumulation is related to the leaf mesophyll conductance and net photosynthesis performed at each provenance. We suggest that in those provenances where *C. quitensis* show a higher mesophyll limitation, CaOx crystals as a carbon source will be used more, and therefore, they will have a lower abundance of crystals in the leaves.

## 2. Results

### 2.1. Morphological Leaf Traits

**Figure 1:**
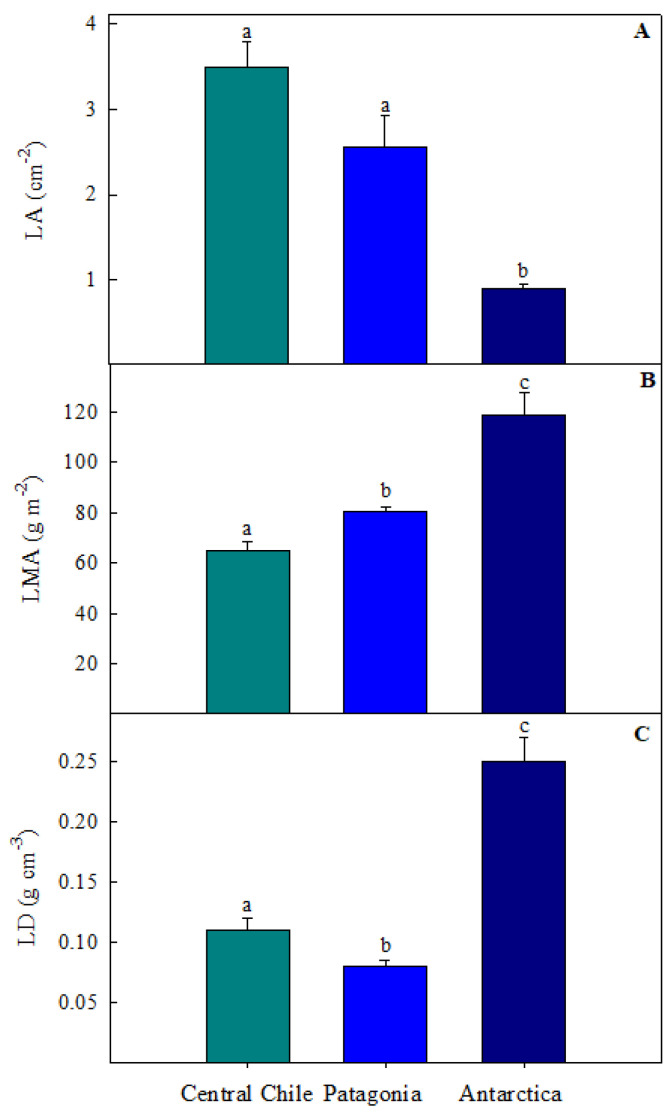
Morphological leaf traits of *Colobanthus quitensis* from three different provenances along the Andean-Patagonian and Antarctic gradient. (**A**) Leaf area (LA), (**B**) Leaf mass area (LMA), and (**C**) Leaf density (LD). Values are means ± S.E. (*n* = 10). Different letters represent statistically significant differences between provenances (Tuckey *p* < 0.05).

*C. quitensis* from different provenances along the Andean (Central Chile), Patagonian, and Antarctic gradient exhibited significant morphological variations in leaf area (LA), leaf mass area (LMA), and leaf density (LD) (Figure 1). Leaf area tended to increase toward lower latitudes, with significant differences among them (Figure 1A). The highest LA value was found in Central Chile (3.4 ± 0.9 cm^2^), while the lowest was recorded in Antarctica (0.9 ± 0.1 cm^2^). On the other hand, LMA and LD tended to decrease toward lower latitudes, also showing significant differences among the provenances, with the highest values in Antarctica (118.65 ± 8.93 g m^−2^ and 0.25 ± 0.02 g m^−3^ for LMA and LD, respectively) and the lowest in Central Chile (65.21 ± 2.99 g m^−2^ and 0.11 ± 0.01 g m^−3^ for LMA and LD, respectively).

### 2.2. Leaf Gas Exchange

**Figure 2:**
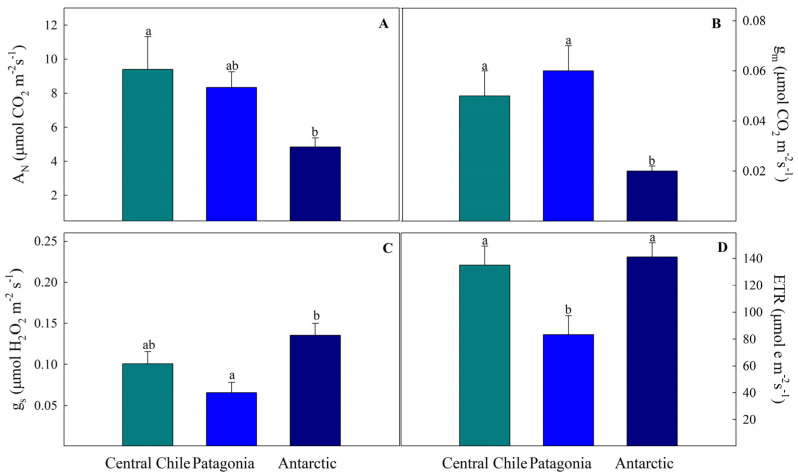
Photosynthetic traits of *Colobanthus quitensis* from three different provenances along the Andean-Patagonian and Antarctic gradient. (**A**), Net photosynthesis at 400 ppm CO_2_ (A_N_), (**B**) Mesophyll conductance (g_m_), (**C**) Stomatal conductance (g_s_), and (**D**) Electron transport rate (ETR). Values are means ± S.E. (*n* = 10). Different letters represent statistically significant differences between provenances (Tuckey; *p* < 0.05).

In the gas exchange parameters, significant differences in A_N_ and g_m_ were observed mainly for plants growing in Antarctica (Figure 2A,B), which showed the lowest values for these parameters (4.85 ± 0.52 and 0.02 ± 0.002 for A_N_ and g_m_, respectively). Although plants from this provenance showed the highest values of stomatal conductance (g_s_), there were only significant differences with the Patagonia provenances (Figure 2C). While the photosynthesis of the Antarctic provenance tended to be lower, the chloroplast electron transport rate (ETR) was high, displaying significant differences when compared with the Patagonia provenance but not with the Central Chile ecotype (Figure 2D).

### 2.3. Leaf CaOx Crystal

**Figure 3:**
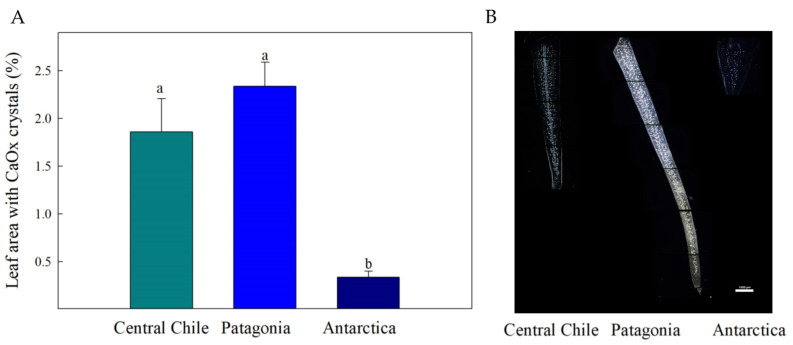
(**A**) The percentage of leaf area occupied by CaOx crystals in *Colobanthus quitensis* from three different provenances along the Andean-Patagonian and Antarctic gradient. Values are means ± S.E. (*n* = 6). Different letters represent statistically significant differences between provenances (Tuckey; *p* < 0.05). (**B**) Representative paradermal view of the chlorine-bleached leaves from three provenances of *C. quitensis* under polarized light (4×). CaOx crystals are visible as bright spots. The white scale bar: 1000 µm.

The percentage of leaf area occupied by CaOx crystals showed significant differences between the Antarctic and the Central Chile andPatagonia provenances (Figure 3). The Antarctic population exhibited the lowest proportion of leaf area occupied by CaOx crystals. In contrast, the Central Chile and Patagonia populations had more leaf area occupied by these crystals, with no significant differences observed between these two provenances.

### 2.4. Relationship between CaOx Crystal and the Anatomical and Photosynthetic Traits

**Figure 4:**
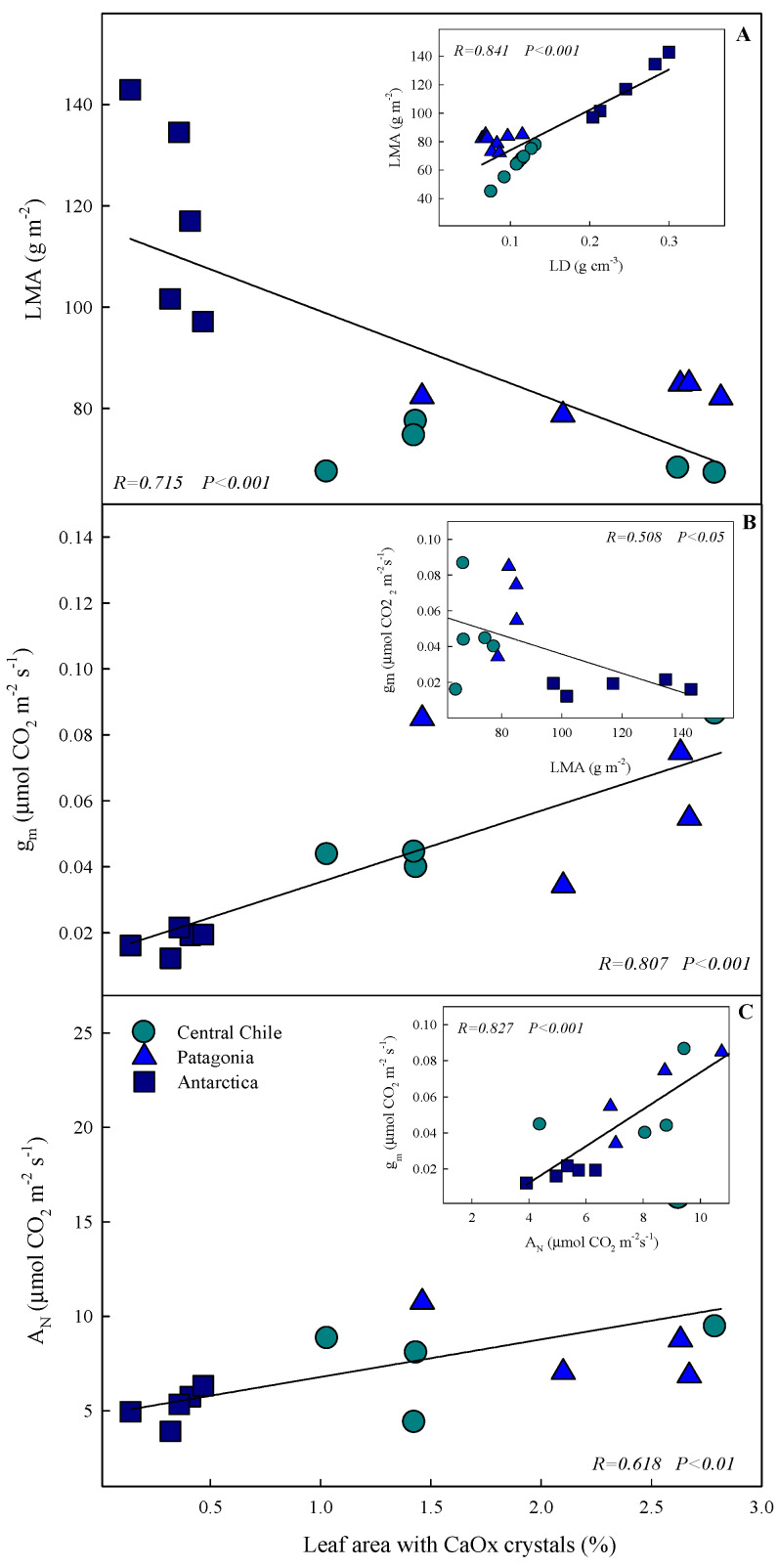
The relationship between the leaf area occupied by CaOx crystals with the: (**A**) Leaf mass area (LMA), (**B**) Mesophyll conductance (g_m_), and (**C**) Net photosynthesis (A_N_) in *Colobanthus quitensis* from three different provenances along the Andean-Patagonian and Antarctic gradient. The graphics inside show the relationship between leaf density (LD) and LMA (**A**), LMA and g_m_ (**B**), and A_N_ and g_m_ (**C**). Regression coefficient and the significance of the relationship are shown considering all provenances together.

The correlation between the percentage of leaf area occupied by CaOx crystals and three key factors—LMA (xerophytic trait), g_m_ (CO_2_ diffusion at leaf level), and A_N_ (photosynthetic performance)—was examined (Figure 4). We found a significant negative correlation between LMA and the percentage of CaOx crystals (R = −0.715, *p* < 0.001); thus, as LMA decreased, the percentage of leaf area occupied by CaOx crystals increased. Lower LMA appeared to be associated with a greater availability of crystals in the leaves. Interestingly, there was a strong positive correlation between the percentage of CaOx crystals and g_m_ (R = 0.807, *p* < 0.001). It appears that at a higher mesophyll, CO_2_ diffusion led to more accumulation of crystals in the leaves. Our analysis also revealed a positive correlation between the percentage of leaf area occupied by CaOx crystals and A_N_ (R = 0.618, *p* < 0.01), indicating that as photosynthesis increased, the plant could accumulate more crystals in the leaves.

## 3. Discussion

CaOX crystal accumulation has been suggested as a biochemical mechanism that contributes to CO_2_ assimilation, especially when the carbon levels for photosynthesis are limited. In *C. quitensis*, the occurrence of CaOX crystals has been described recently as a mechanism that adds to those already described for this species, particularly those associated to the xerophytic leaf traits, which determine the survival of this species in highly hostile climates [9]. In the current study, we evaluate the CaOX crystal accumulation in different provenances of *C. quitensis* within the Andes-Patagonian-Antarctic gradient, relating this accumulation with anatomical and in situ gas exchange parameters. The Antarctic and Central Chile populations have been previously reported as ecotypes for cold resistance. Yet, analysis of the internal transcribed spacer (ITS) region of nuclear ribosomal DNA reveals a relatively high genetic similarity between them despite the significant geographical separation [4]. The Patagonian population is currently under investigation, and this research represents one of the initial studies to include it.

*C. quitensis* from different latitudinal provenances exhibit differences in the anatomical leaf traits, including leaf area (LA), leaf mass area (LMA), and leaf density (LD), all being significantly lower in plants growing in Antarctica (Figure 1). Although the ecological and functional significance of these traits is still under debate [15], it is widely accepted that warmer temperatures are associated with greater leaf expansion and lower leaf thickness. In contrast, higher LMA associated with higher LD is a general response to intense environmental stress, mainly associated with low temperature [15,16,17]. Thus, several studies have reported higher LMA under low winter conditions [18] and low nutrient availability [17]. These agree with previous studies that have evaluated leaf characteristics in plants along a latitudinal gradient in temperate zones, where different plant species consistently exhibit a decrease in leaf size towards higher latitudes [19,20], the same behavior observed in *C. quitensis*.

When we analyze the regional weather conditions in the gradient evaluated, it becomes evident that, in general, the higher latitude (Antarctica) experiences the most severe environmental conditions. Among the climatic factors, temperature and wind speed are two variables that could be influencing the observed physiological traits. Notably, in Antarctica, *C. quitensis* plants are subjected to significantly lower temperatures and higher wind speeds during the study season. However, although some microclimatic conditions could be equally stressing in Andes and Patagonia, what really distinguishes Antarctica from the other provenances is the constant low temperatures during the whole day, even during the entire snow-free period [21]. This could trigger the differences in the leaf traits observed mainly between the Antarctic provenances and the other two provenances.

Such differences are expected to result in stronger mesophyll diffusion limitations in leaves grown in Antarctica. In this line, net photosynthesis (A_N_) and mesophyll conductance (g_m_) tend to decrease towards higher latitudes, being significantly lower in *C. quitensis* growing in Antarctica (Figure 2) and showing a strong correlation between g_m_ and LMA (Figure 4). Despite the impact on net photosynthesis in temperate gradients having received less attention, similar trends have been reported for some species along a gradient. For instance, Elferjani et al. [22] reported that the net photosynthesis decreased or remained unchanged at higher latitudes in *Populus* spp. trees in northern Canada [22]. In Chile, Figueroa et al. [23], studying *Eucryphia cordifolia* spp. over the range of latitudinal distribution (36° to 42° S), showed that despite not observing a consistent pattern, the highest photosynthetic values were observed in plants within populations of intermediate distributions. The authors attribute this variation to the precipitation and temperature gradient [23]. For *C. quitensis*, to our knowledge, this is the first study to evaluate in situ physiological traits associated with gas exchange in the latitudinal distribution of this species. Nevertheless, under controlled laboratory conditions, Acuña-Rodríguez et al. conducted a study on the effect of simulated warming in three populations of *C. quitensis* from a latitudinal gradient, where a clinal trend in the foliar traits of the evaluated *C. quitensis* populations was found. On the other hand, they observed an asymmetric response to warming for the southern populations in all ecophysiological traits evaluated, suggesting that low temperature limits the performance of *C. quitensis* populations [5].

In this sense, at high wind speed and low ambient air temperature, photosynthesis may be reduced due to below-optimal leaf temperatures and stomatal conductance [24,25]. This temperature limitation in *C. quitensis* has been suggested previously by Sáez et al. [12,13], under both field and laboratory conditions, where it was observed that photosynthesis of *C. quitensis* is limited by low temperature, and that increases in temperature induce leaf anatomical changes that result in higher net photosynthesis. In particular, at low temperature, g_m_ strongly limits gas exchange and the availability of CO_2_ at the chloroplast. To cope with this, *C. quitensis* has deployed a compensatory biochemical mechanism based on increasing the specificity of Rubisco (S_c/o_), which contributes to maintaining positive photosynthetic rates [13]. Associated with this, the accumulation of CaOx crystals seems be another biochemical mechanism, acting as an important source of CO_2_ supply for photosynthesis [9]. With this said, the in situ observation of CaOx crystals showed that the percentage of leaf area occupied by CaOx crystals in *C. quitensis* exhibited a significant variation toward the latitudinal gradient (Figure 3), being exceptionally low in the highest latitude. This finding is consistent with our earlier study conducted under common garden conditions, which clearly indicated that the Antarctic population of *C. quitensis* shows less crystal accumulation in leaves compared to Andean and Patagonian populations [14].

Our study highlights a novel relation between alarm photosynthesis and mesophyll conductance to CO_2_ diffusion in *C. quitensis*, elucidating the possible ecological role of CaOx crystals in this process. The observed positive correlations between the percentage of leaf area occupied by CaOx crystals with both g_m_ and A_N_ may suggest that CaOx crystals are integral to optimizing CO_2_ diffusion and enhancing photosynthetic efficiency, particularly under environmental stress. This proposed adaptive strategy, most evident in the Antarctic provenance, highlights the plant’s physiological plasticity and its putative ability to utilize CaOx crystals as an alternative carbon source, shedding light on the complex mechanisms vascular plants employ to thrive in extreme environments. These results are consistent with our hypothesis that in the Antarctic provenance, where *C. quitensis* deploys stronger xerophytic traits (i.e., higher LMA and LD), we also observed the highest mesophyll limitation, and a lower abundance of CaOx crystals. As CaOx crystals in *C. quitensis* are a dynamic system and fluctuate in the course of a day (with full recovery during night hours) [9], we suggest that the lower abundance is associated with higher use of CaOx crystals as a carbon source. Thus, a stronger correlation was found between the leaf CaOx crystal accumulation and LMA, g_m_, and A_N_ performed in situ (Figure 4).

While our findings offer significant insights into plant adaptation mechanisms, we recognize the preliminary nature of our results. The correlations observed suggest a potential mechanism by which CaOx crystals may contribute to the plant’s adaptation to extreme environments, but we caution that these results are initial steps towards fully understanding the role of CaOx in carbon assimilation and storage, highlighting the necessity for direct experimental evidence to more firmly establish the relationship between CaOx crystal accumulation, mesophyll conductance, and photosynthetic efficiency. Our study serves as a foundation for future research, proposing a possible interaction that warrants deeper exploration with more direct methods to clarify the role of CaOx crystals in enhancing plant tolerance to extreme conditions.

## 4. Materials and Methods

### 4.1. Study Sites and Plant Collection

**Figure 5:**
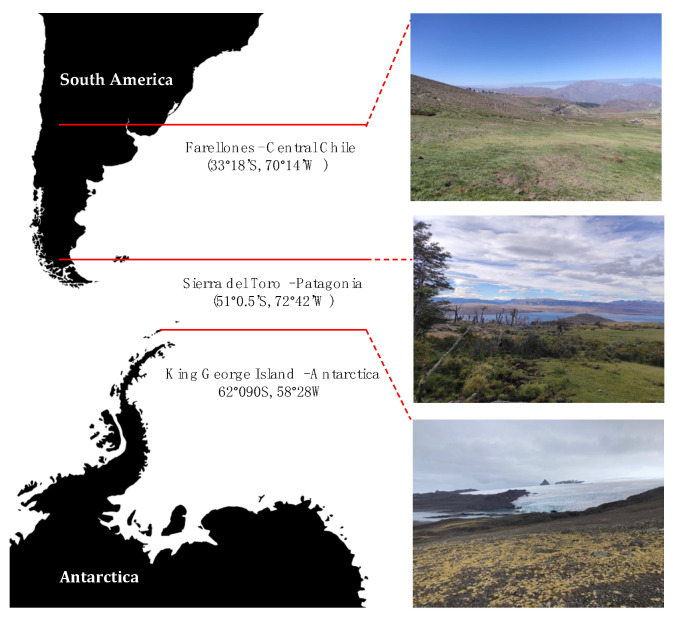
Geographical locations and landscape view of *C. quitensis* provenance sampling sites.

*C. quitensis* from three sites within a latitudinal gradient (≈3350 km, from Andes mountains in Central Chile to Antarctica, Figure 5) were selected for in situ gas exchange measurements and leaf samples collection. The sites were as follows: Farellones in Central Chile (Lo Barnechea, Chile—33°18′ S, 70°14′ W), Sierra del Toro in Patagonia (Torres del Paine, Chile—51°0.5′ S, 72°42′ W), and King George Island in Antarctica (Henryk Arctowski Polish Antarctic Station—62°090 S, 58°28 W).

Central Chile is characterized by a Mediterranean-type climate, with marked summer drought that mainly influences low elevations [26]. The study site in Patagonia is immersed in the Subregion of the Patagonian Steppe of Magallanes [27], which corresponds to a homogeneous vegetation, where shrubs, caespitose herbs, and grasses predominate. Finally, the site in Antarctica corresponds to the study site described by Sáez et al., 2018 [13] in the vicinity of the Henryk Arctowski Polish Antarctic Station, on King George Island, off the coast of Antarctica. Detailed environmental conditions from the three regions can be found in Table 1.

### 4.2. Leaf Mass Area and Leaf Density

Leaf mass per area (LMA) was obtained by comparing the dry weight of the leaf to its surface area. To achieve this, ten plants from each origin were chosen at random, with a minimum of six leaves sampled from each plant for evaluation. The area of fresh leaves was assessed using ImageJ software version 1.54g (Wayne Rasband/NIH, Bethesda, MD, USA). The leaves were then dehydrated in an oven at 70 °C for 64 h to ascertain their dry weight. Additionally, leaf density (LD) was calculated by dividing the LMA by the thickness of the leaf, which was measured from leaf cross-sections using an optical microscope (Euromex iScope, Arnhem, The Netherlands).

### 4.3. Leaf Gas Exchange and Chl Fluorescence

Leaf gas exchange and chlorophyll a fluorescence were assessed using a Li-6400XT, with a Li-6400-40 leaf chamber (Li-Cor Inc., Lincoln, NE, USA) following the methodology outlined by Sáez et al. [12]. Measurements were conducted on several leaves per specimen to fully utilize the chamber area of the infrared gas analyzers without overlapping the leaves. Corrections for measurements were made based on the estimated leaf area within the chamber. The leaf’s temperature was recorded via a thermocouple (6400-04, Li-Cor Inc.) placed on the leaf’s underside. These gas exchange readings were taken at a constant leaf temperature of 15 °C.

The quantum yield of photosystem II (PSII) electron transport was calculated using the following: ϕPSII = (F′_m_ − F_s_)/F′_m_, where Fs is the light-adapted steady-state fluorescence (PPFD 1000 μmol photons m^−2^ s^−1^) and F′_m_ is the maximum fluorescence from a saturating pulse of light (8000 μmol photons m^−2^ s^−1^). Since ϕPSII indicates the electrons moved per photon absorbed by PSII, the electron transport rate (ETR) was derived as follows: ETR = ϕPSII • PPFD αβ, incorporating the photosynthetic photon flux density (PPFD), leaf absorptance (α), and the assumed equal distribution of absorbed energy between photosystems (β, set at 0.5). Leaf absorptance, measured as in Sáez et al. [17], showed values of 0.93 ± 0.01, 0.94 ± 0.01, and 0.89 ± 0.001 for plants from Central Chile, Patagonia, and Antarctica, respectively. The mesophyll conductance to CO_2_ (g_m_) was determined following Harley et al.’s formula [18]:

g_m_ = A_N_/(C_i_ − (Γ × (ETR + 8 (A_N_ + R_L_))/(ETR − 4 (A_N_ + R_L_)))) (1)

with A_N_ and C_i_ derived from gas exchange data at saturating PPFD (1000 µmol photons m^−2^ s^−1^). The rate of non-photorespiratory CO_2_ evolution in the light (R_L_) was assumed to be half of R_dark_, and the chloroplast CO_2_ compensation point (Γ*) was determined based on Brooks and Farquhar’s method using the in vitro measured Rubisco specificity factor [NO_PRINTED_FORM] (S_c/o_) [13].

### 4.4. CaOx Crystal Measurements in the Leaves

Measurements of the CaOx crystal areas within the leaves followed the protocol outlined by Gómez et al. (2020) [9]. In summary, leaves were first bleached using a 5% sodium hypochlorite solution, immersing them completely for 48 h to achieve total depigmentation. After bleaching, the leaves were thoroughly washed with distilled water and then sandwiched between two glass slides to ensure they were fully spread out. Observations were made with a light microscope equipped with a polarizing filter at a 10× enlargement, using a Euromex CMEX camera and a Euromex iScope (Euromex Microscopen bv, Papenkamp, The Netherlands). Multiple photographs were captured across the entirety of each leaf’s surface. Digital image analysis software (ImageJ-Fiji v 2.0.0-rc69/1.52i) [28] was employed to calculate the area occupied by each crystal. The sums of all crystal areas from a single leaf were compiled to determine the leaf’s total crystal area, which was then normalized to the leaf’s surface area to calculate the ratio of crystal area to leaf area.

### 4.5. Statistical Analysis

The effects of provenance on the leaf anatomy, photosynthetic performance, and CaOx crystal accumulation were assessed through ANOVA (*p* = 0.05), and differences among means were determined with Tukey-test (*p* < 0.05). Analyses were performed with the InfoStat/L [29]. A Pearson correlation analysis was performed to assess the relationships of the different anatomical and photosynthetic traits with CaOx crystal accumulation, all of which were carried out using SigmaPlot Version 10.0.

## 5. Conclusions

Our study provides evidence that the dynamics of CaOx crystal accumulation and decomposition in *C. quitensis* are closely tied to the plant’s adaptation strategies in response to extreme environmental conditions, particularly temperature variations along the Andes-Patagonian-Antarctic gradient. This research marks the first in-depth examination of in situ physiological traits related to gas exchange across *C. quitensis* latitudinal distribution. Notably, our findings reveal a significant variation in anatomical leaf traits such as leaf area, leaf mass area, and leaf density, with the most pronounced xerophytic traits observed in Antarctica. These traits correlate strongly with reduced mesophyll conductance and net photosynthesis in Antarctic populations, suggesting a more pronounced leaf mesophyll limitation in these plants. Thus, this study extends our understanding of *C. quitensis’* adaptation mechanisms, highlighting the role of temperature as a critical factor influencing leaf morphology and photosynthetic efficiency. The observed decrease in CaOx crystal abundance in Antarctic *C. quitensis*, alongside a higher dependence on these crystals as a key carbon source due to increased mesophyll limitations, aligns with our hypothesis. Our findings evidenced the adaptation in leaf traits and the biochemical use of CaOx crystals to maintain efficient photosynthesis under hostile climatic conditions. Further research should be performed in order to establish a mechanistic relationship between CaOx crystal accumulation and photosynthetic performance, mainly mesophyll conductance, and to clarify the contribution of CaOx as an internal source of carbon, and the whole biochemical pathways involved in alarm photosynthesis in *C. quitensis.*

## Figures and Tables

**Table 1:** Monthly weather conditions near the study sites for the summer period.

	El Colorado Station (Lo Barnechea)—Central Chile	Río Serrano Station (Torres del Paine)—Patagonia	C.M.A. Eduardo Frei Montalva Station—King George Island
Average temperature (°C)	10.5	8	0
Max temperature (°C)	25	20	2
Min temperature (°C)	5	3	−4
Total Precipitation (mm)	0.2	5	45
Solar radiation kwh/m²	13,180.6	7467.5	-
UV Index	4	-	4
Light Hours	13.5	14.6	18.5
Average wind speed (m/s)	5.1	6.4	10.2
Average relative humidity (%)	51.5	75	95

Source: Chile’s National Weather Service.

## Data Availability

Data are contained within the article.
